# Interfacial Water Stability between Modified Phosphogypsum Asphalt Mortar and Aggregate Based on Molecular Dynamics

**DOI:** 10.3390/polym15224412

**Published:** 2023-11-15

**Authors:** Cancan Liang, Yilang Li, Ponan Feng, Yuanle Li

**Affiliations:** 1 College of Mechanical & Electrical Engineering, Henan Agricultural University, Zhengzhou 450002, China; 15036016125@163.com; 2 Nan County Tobacco Monopoly Bureau, Yiyang 413299, China; liyl970527@163.com; 3 School of Highway, Chang’an University, South Erhuan Middle Section, Xi’an 710064, China; lyl199611@126.com

**Keywords:** water stability, modified phosphogypsum, coupling agents, asphalt mortar, interfacial behaviour, molecular dynamics

## Abstract

The objective of this study is to unravel the modification mechanism of a coupling agent on the water sensitivity of phosphogypsum asphalt mortar. The adhesion process of phosphogypsum asphalt mastic modified with three kinds of coupling agents (KH-550, KH-570, and CS-101) and raw phosphogypsum to the aggregate minerals was simulated based on the molecular dynamics software, Materials Studio 2020, and the water film layer was considered along the simulation. When the three coupling agents were added, the interfacial adhesion work gradually increased with the increase of modified phosphogypsum dosage, and the trends of each model were relatively similar. With the increase of simulation time, the mean square displacement of water molecules of the three interfacial models showed different trends, and the increasing trend rank was unmodified phosphogypsum > KH-550 > KH-570 > CS-101. The diffusion coefficient of the water molecular layer of asphalt mastic modified with CS-101 coupling agent in phosphogypsum shows a significant decrease with the increase of CS-101-modified phosphogypsum (more than 5% mass ratio to asphalt). Compared to raw phosphogypsum asphalt mortar, the addition of coupling agents can significantly limit the diffusion of water molecules and effectively improve the interfacial adhesion work, in which CS-101 coupling agent has the best effect, followed by KH-570 and KH-550.

## 1. Introduction

Phosphogypsum, as a kind of solid waste from the chemical industry, is frequently used as a modifier and sustainable filler for asphalt materials, and often adopted to produce phosphogypsum asphalt mortar (PAM). Due to the intricate composition and surface structure of phosphogypsum, direct application to pavement is usually not feasible [1]. Therefore, it is necessary to pre-treat and modify it appropriately for better suitability and performance. Due to the large volume and wide application of asphalt mixtures, applying phosphogypsum as a filler instead of traditional mineral powder filler in the asphalt mixture of a road project can not only solve the problem of shortage of natural minerals, but also promote the regeneration and utilisation of phosphogypsum waste resources, which has a wide range of social and economic benefits [2,3]. Disposing of the trace elements present in phosphogypsum poses a challenge, impeding its use in road base soils [1] while full-scale experimentation of phosphogypsum application in asphalt materials for pavements is currently ongoing [1,4]. In order to ensure that its water stability approaches that of generic asphalt mixtures, a silane coupling agent is usually selected as an external dopant to modify the phosphogypsum. However, coupling agent modification of phosphogypsum is essentially a chemical modification process, and the products of various types of coupling agents reacting with phosphogypsum are the main factors affecting the water stability of phosphogypsum mortar. Recently, researchers have primarily focused on examining the high temperature stability and other mechanical traits of PAM, with only a few investigations into its water stability [4].

To examine the influence of phosphogypsum asphalt properties, researchers added varying amounts of phosphogypsum to asphalt to explore the impact of phosphogypsum filler on enhancing asphalt pavement performance. The study revealed that incorporating phosphogypsum onto asphalt can enhance its high-temperature rheological characteristics without compromising the low-temperature properties. Additionally, it increases viscosity and the softening point while reducing penetration levels [5]. Diao et al. concluded that the addition of phosphogypsum reduces the temperature sensitivity of asphalt while hardening it, but does not improve the low temperature properties of asphalt [6]. Qiu et al. measured the optimal asphalt-to-phosphogypsum powder ratio and observed that this ratio has an impact on properties such as tensile strength and service life. The results suggest that careful consideration should be given to the ratio when designing asphalt mixtures. Further research into the effects of different ratios on asphalt composition is recommended [7]. Cuadri et al. examined the potential use of phosphogypsum as an asphalt modifier through Fourier transform infra-red analysis. Their findings revealed that the blend is a chemical reaction [8]. Viscosity flow experiments carried out by Cuadri et al. on PAM augmented with sulfuric acid revealed a marked rise in viscosity at 60 degrees Celsius relative to gypsum-modified samples [9]. Kim et al. discovered that incorporating a mineral additive composed of 7% desulfurized phosphogypsum into cold recycled asphalt mixtures fulfilled the modulus demands for pavement materials in Korea, as the mixing of inorganic materials had a relatively small effect on temperature sensitiveness of the overall binder [10]. Li et al. analysed the impacts of varied doses of phosphogypsum calcium sulfate whiskers on the physical features of matrix asphalt. They discovered that an appropriate number of whiskers can efficiently enhance the high-temperature properties of asphalt, but it also adversely affects its low-temperature performance. The ideal quantity is between 5% and 9% of the asphalt content [11].

Li et al. discovered that adding a suitable quantity of steel slag powder can enhance the softening point of phosphogypsum asphalt mortar, bolster its high-temperature capabilities, and highly augment the adhesion and water stability of PAM for its water stability [12]. Ignatiev et al. demonstrated that the incorporation of polyethylene terephthalate and industrial waste phosphogypsum into the dispersant can enhance the water resistance of asphalt concrete. Meanwhile, some scholars investigated the application of waterproofing agents for phosphogypsum materials or mineral–asphalt interface [13].

Chen et al. discovered that incorporating silane coupling agent (SCA) A151 and polyvinyl alcohol composite waterproofing agents had a profound positive impact on the waterproofing performance of phosphogypsum products [14]. Zhang et al. previously reported that the use of titanate ester coupling agent (TECA) can enhance the interface performance of granite aggregates. Specifically, when the concentration of TECA in asphalt reaches 0.37%, the adhesion level improves from level two to level five [15]. Liu et al. discovered that SCA at levels between 0.5% to 2.5% leads to substantial enhancements in the thermal and aqueous stability of asphalt mixtures utilising granite as an aggregate. Peng et al. concluded that with the increase of SCA, the surface free energy and FTIR parameters basically increased initially, reached a maximum at 1.0 wt% and then decreased, indicating that SCA can act as a molecular bridge to improve the adhesion properties between acidic aggregate and asphalt [16]. Cao et al. employed molecular dynamics simulations to unveil that the γ-aminopropyltriethoxysilane (KH-550) SCA-modified system possesses the greatest absolute value of interaction energy, suggesting that it has the most potent impact in enhancing the interfacial adhesion amidst asphalt and iron tailings [17].

In summary, PAM may suffer from inadequate water stability. However, incorporating waterproof modifications of phosphogypsum and adhesion modifications of asphalt aggregate indicates that SCA and TECA could be viable interfaces to enhance water stability during the modification of asphalt with phosphogypsum.

To unveil the reason behind the insufficient water stability of PAM, traditional test methods cannot provide the necessary targeted research into the microscopic molecular structure and interfacial adhesion. Therefore, the molecular dynamics (MD) simulation method was employed, utilizing Materials Studio (MS) 2020 commercial software. The molecular models for PAM binder modified by common coupling agents (including SCA and TECA) as well as mineral aggregate were established, respectively. With the assistance of this process, the adhesion of PAM binder with aggregate minerals in the presence of water was analysed. The interfacial mechanism, which involves the adhesion function and interfacial water diffusion behaviour of the coupling agent modification mechanism, will be calculated to investigate the role of the coupling agent in surface modification for phosphogypsum in the asphalt-based binder system. This analysis will ensure a comprehensive understanding of the interfacial process.

## 2. Materials and Methods

To examine the fundamental cause of differing adhesion abilities between asphalt and aggregates of different mineral components, dynamic calculations were carried out to investigate interface interactions between each mineral component and asphalt in depth. To simulate and investigate the process, asphalt and aggregate mineral models were constructed firstly.

### 2.1. Construction of Asphalt Mortar

#### 2.1.1. Matrix Asphalt Components

For the matrix asphalt model, components, numbers and respective ratios were based on the 12 molecule AAA-1 asphalt model proposed by Li et al. [18], as shown in Table 1. The Amorphous Cell module of MS was employed to mix each molecular component in the system according to the corresponding amount to represent the matrix asphalt.

**Figure 1:**
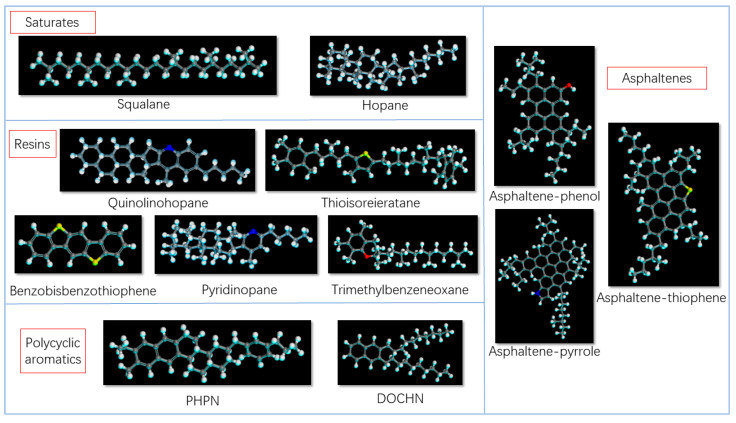
Models of asphalt components.

As the main research focus is the PAM mortar model, a larger matrix asphalt system was required for the mortar model. Drawing from relevant studies on mortar model construction, and to facilitate control of phosphogypsum components mixed into the asphalt model with varying mass ratios, the matrix asphalt components in Table 1 were doubled to construct the matrix asphalt model. Furthermore, this section presents the procedure for constructing matrix asphalt components. Firstly, the molecular structure models of the molecules listed in Table 1 are drawn in Visualizer [18], and the “Clean” operation is performed to keep them in a reasonable spatial state. Then, 5000 iterations of geometric optimization are performed on each component, and the other parameters in the optimization process are default values. The model results are shown in Figure 1 (where the white atoms are hydrogen, black atoms are carbon).

#### 2.1.2. Phosphogypsum Molecular Model

**Figure 2:**
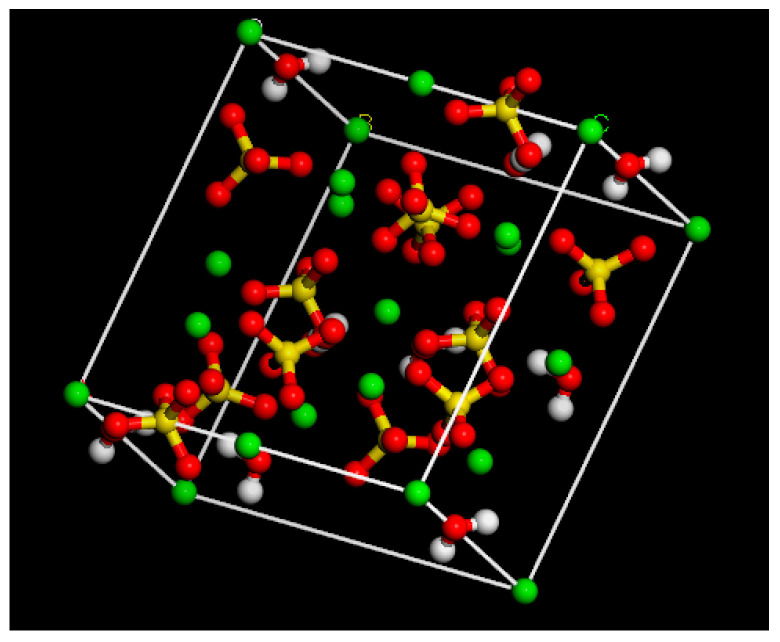
Crystal model of calcium sulfate hemihydrate.

According to relevant research, the primary constituent of phosphogypsum material is calcium sulfate hemihydrate (CaSO_4_·½ H_2_O), which belongs to the monoclinic system [19]. The SO_4_^2−^ tetrahedron and Ca^2+^ ions are linked in parallel layers along the b–c crystal axis. The coordination number of Ca^2+^ is six, and the SO_4_^2−^ tetrahedron and Ca^2+^ are connected in a chain shape in the c-axis direction. There are pores situated between the chains wherein H_2_O is located and shared by two adjacent crystal cells. This H_2_O is connected to the O^2−^ in SO_4_^2−^ via hydrogen bonding. Consequently, a crystal model of hemihydrate calcium sulfate is constructed based on this feature, which is illustrated in Figure 2 (where the white atoms are hydrogen, red atoms are oxygen, yellow atoms are sulphur, and green atoms are calcium).

**Figure 3:**
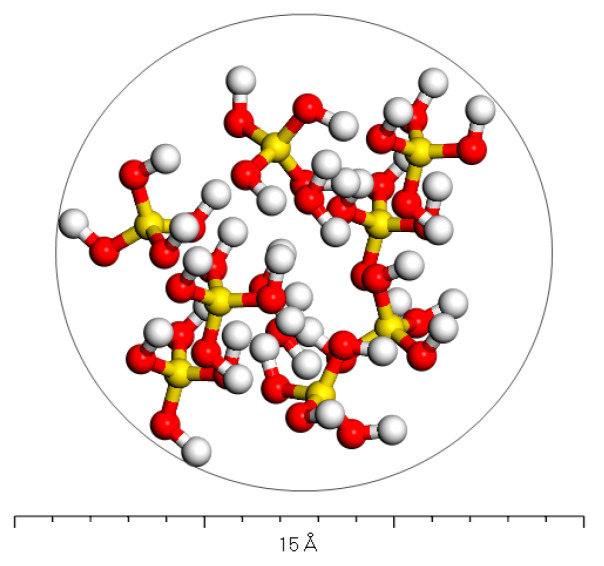
Nanocluster model of hydroxylated hemihydrate calcium sulfate.

Based on the crystal model of calcium sulfate hemihydrate and considering the model construction principles of molecular simulations related to asphalt mortar research, a molecular model of phosphogypsum filler components has been developed with the use of nanoclusters, based on the steps shown in Section S1 of the Supporting Information. According to related research, unlike aggregate minerals, after hydroxylation of phosphogypsum, the calcium ions of hemihydrate calcium sulfate are filtered out of the solution in a free state, so the remaining material is mainly hydroxylated sulfate ions [20,21]. Then, the removal of calcium ions is carried out. The simulation shown in Figure 3 operated based on this model (where the representation of atomic colours is the same as above).

#### 2.1.3. Construction of Coupling Agent Model

**Figure 4:**
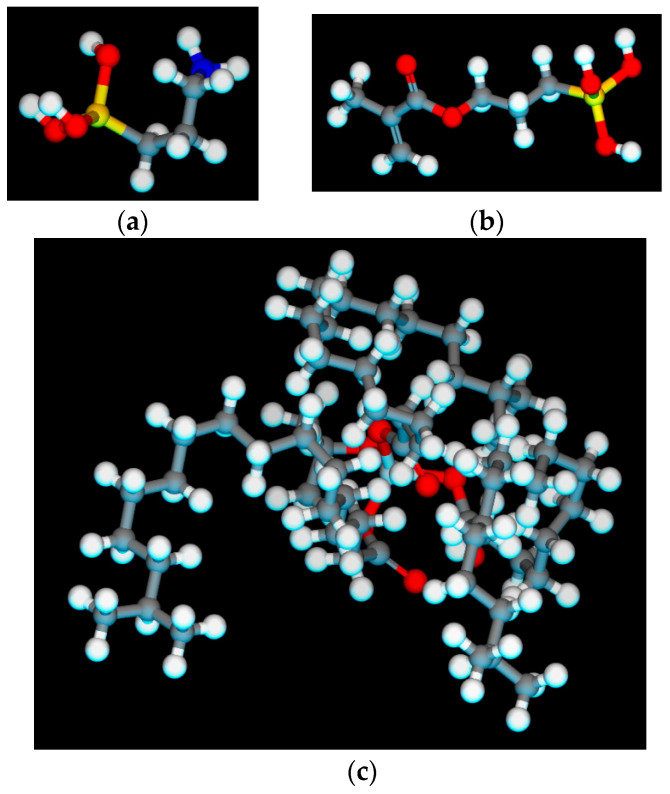
Molecular models of three coupling agents after hydrolysis: (**a**) KH-550; (**b**) KH-570; (**c**) Titanate CS-101.

According to the research of relevant scholars [22,23], Si-O-CH_3_ in the molecular structure of the silane coupling agent can be combined with the surface groups of inorganic materials through hydrolysis. Thus, it is imperative to initially consider the molecular structure of the three variants of silane coupling agents subsequent to hydrolysis. The molecular models of silane KH-550, γ-methacryloxypropyltrimethoxysilane (KH-570), and monoalkoxytitanate (CS-101) used in this article after hydrolysis were directly constructed in Visualizer, as shown in Figure 4 (where the blue atom is nitrogen element, and the colour representation of other atoms is the same as above).

#### 2.1.4. Assembly of Asphalt Mortar

**Figure 5:**
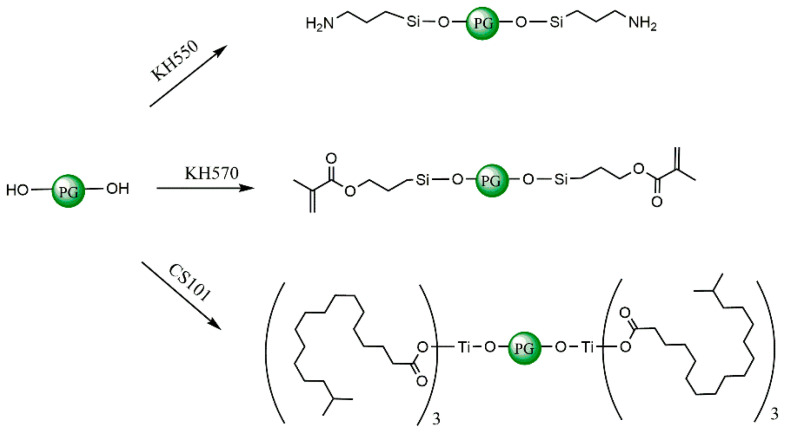
Mechanism diagram of coupling agent-modified phosphogypsum.

**Figure 6:**
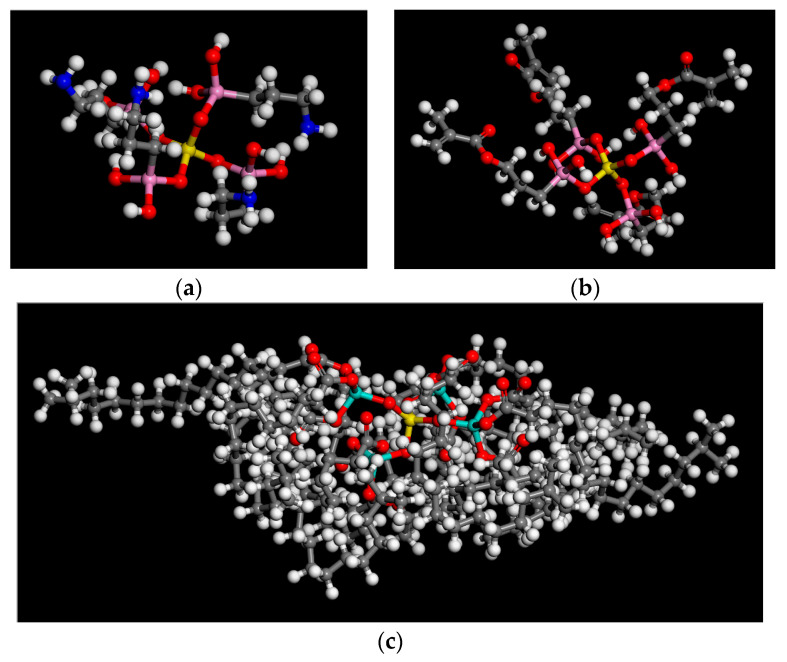
Simplified molecular models of coupling agent-modified phosphogypsum: (**a**) Product of KH-550; (**b**) Product of KH-570; (**c**) Product of titanate CS-101.

Firstly, the reaction principles of three phosphogypsum–modified coupling agents were used in combination with the above molecular model. Based on the relevant research, the coupling agent and the chemical grafting mechanism of phosphogypsum can be simplified and displayed as depicted in Figure 5, where the PG represents various types of phosphogypsum [23]. Hydroxylated hemihydrate calcium sulfate was the primary component employed to build the reaction products of the three coupling agents of chemically modified phosphogypsum, as depicted in Figure 6, where the silicon atoms are specifically coloured pink and the titanium atoms are coloured celeste. To enable a comparative analysis of the effects of modified phosphogypsum content on the water stability of asphalt mortar, we calculated the mass percentage of three coupling agent-modified phosphogypsum fillers in the asphalt mortar system based on the relative molecular weight of the reaction products. Combined with the mass fractions adopted in relevant studies, PAM molecular models with four different mass ratios of mineral powder to asphalt (MRPA) were created (1%, 5%, 10%, and 15% with tolerance range less than 0.3%). Due to the high molecular weight of CS-101 titanate, the MRPA mentioned earlier was chosen based on mass ratios calculated from PAM with varying numbers of CS-101 molecules.

**Figure 7:**
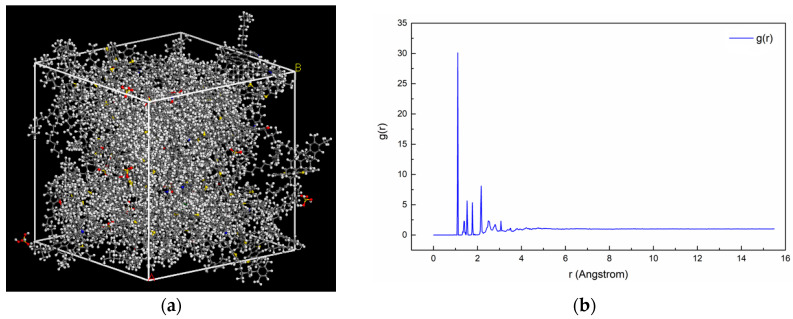
Completed asphalt mortar model and RDF analysis results: (**a**) Initial phosphogypsum asphalt mortar model; (**b**) RDF of the constructed asphalt mortar model.

Through the approach in Section S1 of the Supporting Information, the asphalt mortar model was built and preliminarily analysed, as shown in Figure 7. Figure 7a displays the initial phosphogypsum asphalt mortar model following quench and geometric optimization, and the completed model aligns closely with models in related studies [24]. Through analysing the radial distribution function (RDF) of the constructed model, the rationality of asphalt mortar model was analysed. The results were obtained based on Equation (1), which provides the theoretical basis for RDF calculation, as displayed in Figure 7b. According to the relevant research, the RDF analysis of the molecular model of asphalt-like materials can characterise the rationality of the model [21,25]. The theoretical basis is that the atoms of bituminous materials are disordered in the crystal lattice, so the RDF should be stable at a certain value when the interparticle distance is large. Figure 7b shows that the RDF results are stable around 1 when the inter-particle distance is more than 4 Å, indicating that the model of this asphalt mastic has a preferable rationality. This model will be employed for further calculations in subsequent related studies, based on the aforementioned analysis and construction [26].

(1) $$g(r)=\frac{dN}{\rho 4\pi r^{2}dr}$$

where the *N* represents the total number of particles in the system; *ρ* is the system density (kg/m^3^); *R* is the distance between particles (m).

### 2.2. Construction of Interfacial Model between PAM Mortar and Aggregate Mineral

#### 2.2.1. Aggregate Mineral Molecular Model

**Figure 8:**
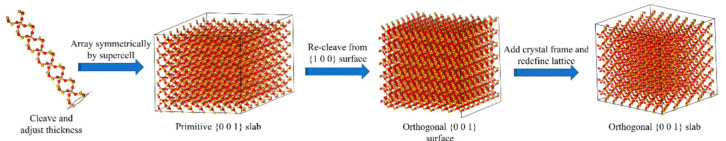
Construction process of orthogonal aggregate mineral model.

The prevalent quartz mineral composition applied in road coarse aggregate was utilized, and α-Quartz was selected as the prototype for quartz crystal cells from the MS 2020 software structure library. Based on the research progress of diagenetic minerals and Chu et al.’s research, α-Quartz is the molecular model construction basis for mineral aggregates [27]. We utilised the “Clean” operation of the “Modify” function on various Miller index surfaces for slicing, followed by utilization of the “Build” tool to generate a combined surface model with crystal planes during the model construction process [25]. The specific steps include:

1. The appropriate thickness of α-Quartz primary cells in the resource library of the MS software system is determined by cleaving them with the Clear function within the range of 6.3 ± 0.6 Å. The determination of the suitable threshold is founded on the premise that the molecular configuration of the rear of the “Cleave” lacks any atoms that are isolated or linked by a lone chemical bond.
2. The supercell function is utilized to perform array replication on the surface of the initial cleavage, resulting in the construction of a supercell based on the supercell parameters setting of u = 9 and v = 9, respectively.
3. The non-orthogonal mineral composition model undergoes secondary sectioning through the “Reclave” operation. The selection of the profile here is based on [27]. After completion of the “Reclave” procedure, a 10 Å vacuum layer is added to the layered model to achieve the orthogonal aggregate model. Subsequently, the Miller surface of the construction target should be adjusted to face upward through the setting of crystal cell parameters, as depicted in Figure 8 (where the colour representation of atoms is the same as above).

#### 2.2.2. Water Molecule Model

Chemical modification may be employed to offset the impact of phosphogypsum incorporation on the bond between aggregate and asphalt, and bolster water stability via the inclusion of coupling agents. Therefore, the MS research considered the influence of the water film on the interface between asphalt mortar and aggregate to analyse the modification effect of various coupling agents on the water stability of PAM. Firstly, a standard TIP3P water molecule model was introduced, and the “Amorphous Cell” module was used. The required quantity of water molecules was altered to create two varieties of water films, each with a thickness of 5 Å and a target density of 1 g/cm^3^, which was accomplished by utilizing the axial lengths of mineral aggregate and asphalt crystal cell models and carrying out appropriate geometric optimization procedures.

#### 2.2.3. Interface Model between PAM Mortar and Aggregate Mineral Model

**Figure 9:**
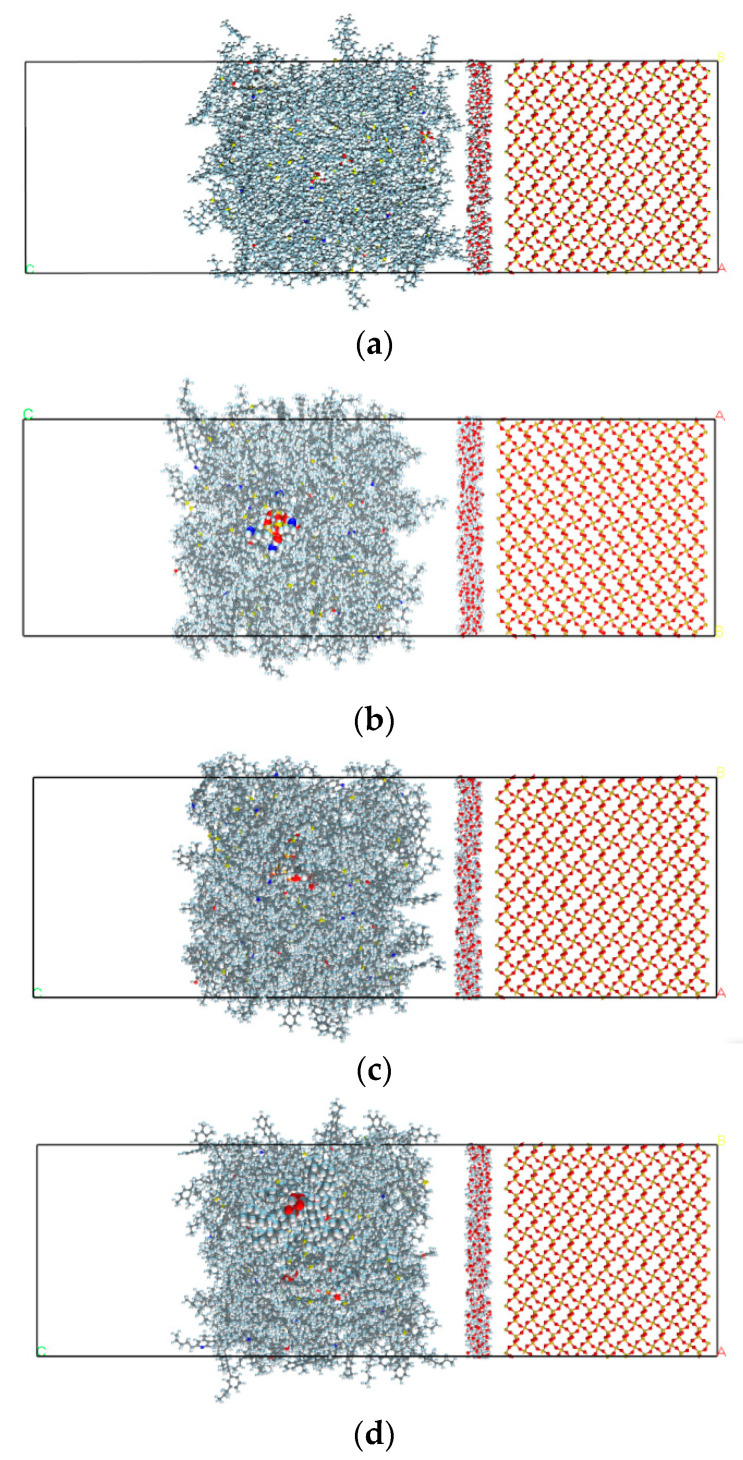
Interface molecular model of asphalt mortar–water–aggregate with phosphogypsum and different coupling agent-modified phosphogypsum: (**a**) PAM based on unmodified phosphogypsum; (**b**) PAM based on KH-550-modified phosphogypsum; (**c**) PAM based on KH-570-modified phosphogypsum; (**d**) PAM based on CS-101-modified phosphogypsum.

After all the models were separately constructed, the interface model was finally built based on the procedure in Section S1 of the Supporting Information. The selection of model size is determined by the crystal parameters of aggregate minerals. To enable the observation of coupling agent components, the relevant molecular structure is displayed using the Corey–Pauling–Koltun model, illustrated in Figure 9. In Figure 9, each model includes three layers, with different types of PAM models on the left, a water molecule model in the middle and a mineral aggregate model on the right constructed by the method shown in Figure 8. The dynamics calculations of each model are then conducted to analyse the adhesion process of PAM and aggregate when there is influence of water.

## 3. Result Analysis

### 3.1. Adhesion Work of PAM–Water–Aggregate Interface

According to the dynamics calculation described in Section S2 of the Supporting Information, further analysis is summarised as follows. For the simulations of the interface between the phosphogypsum filler asphalt mastic and aggregate, considering the presence of a water film, we calculated the adhesion work of the water model using Equation (2). This allows for a more direct expression of the effect of the water film on the interfacial behaviour, taking into consideration the different cross-sectional areas of the models. In the presence of water, the energy of the system will alter following the calculation because of the presence of a water film. In light of this alteration, the impact of the interfacial model system that accounts for the presence of the water film can be quantified through the use of W_mwa_ in Equation (2), where the energy conversion factor is referenced from the energy conversion table proposed by the University of California and the National Institute of Standards and Technology [28].

W_mwa_ = [(E_m_ + E_a_ + E_w_ − E_mwa_) × 6.95 × 10^−21^]/S (2)

where W_mwa_ (mJ/m^2^) represents the work of adhesion for the interfacial model considering water; E_m_ and E_a_ represent the potential energy of the mineral composition model and the bituminous mastic model, respectively; S (Å^2^ = 10^−20^ m^2^) is the interfacial contact area of the model; E_w_ represents the potential energy of the water layer; and E_mwa_ (kcal/mol) represents the total potential energy of the entire modelled system including the corresponding water layer.

**Figure 10:**
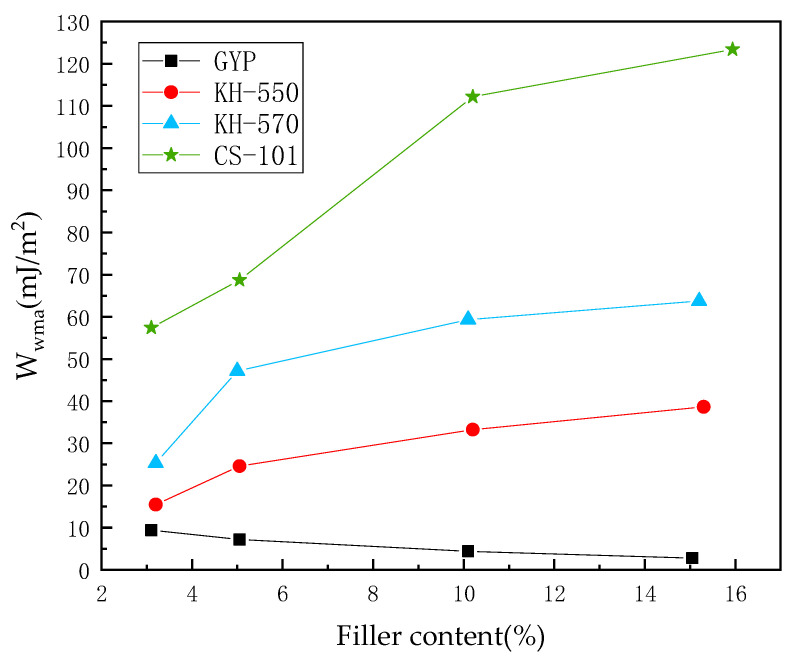
Adhesion work of PAM–Water–Aggregate interface under different filler contents.

To represent the control group of asphalt mastic in different ratios of coupling agent mineral powder to asphalt mass, we used unmodified phosphogypsum and phosphogypsum modified by the three corresponding coupling agents in the control group model, which were phosphogypsum (GYP), KH-550, KH-570 and CS-101, respectively. The calculations were then plotted in Figure 10.

As depicted in Figure 10, the absence of a coupling agent results in a detrimental impact on the interfacial adhesion between asphalt and aggregate. The introduction of phosphogypsum filler exacerbates this negative effect. With increasing dosages of the filler, ranging from approximately 3% to 15%, there is a prominent reduction in W_mwa_. The results indicate that the hydroxylated sulfate ions present in the phosphogypsum exhibit high hydrophilicity which, in turn, leads to faster diffusion of small molecules in asphalt. This significantly affects the adhesiveness of the asphalt to the aggregate in the presence of a water film. The presence of a water film impacts the adhesion between asphalt and aggregate. Various coupling agent components can enhance asphalt mastic–aggregate mineral adhesion. For example, coupling agent-modified phosphogypsum has shown a positive effect. As the quantity of filler increased, W_mwa_ showed a marked trend of improvement, indicating further enhancement after coupling agent modification. The molecular chain of phosphogypsum significantly expands. The initial hydrophilic sulfate group is transformed into the lengthy carbon chain of the coupling agent, simplifying the entanglement of macromolecular chains with asphalt molecules. It is possible to observe the phenomenon of entanglement or macromolecular group agglomeration with asphalt molecules, leading to the adsorption of asphalt molecules and mineral components of aggregates.

### 3.2. Analysis of the Degree of Diffusion of the Water Molecule Layer

In the molecular dynamics simulation environment, individual molecules are in constant thermal motion. The mean square displacement (MSD) measures the average of the squares of the atomic displacements in the system at any given moment. Through MSD calculations, the diffusion pattern of moisture on the mineral surface can be analysed. Equation (3) presents the computation of MSD.

(3) $$MSD\left(t\right)=\frac{1}{N}\sum_{i=1}^{N}{\left[r_{i}\left(t\right)-r_{i}\left(0\right)\right]}^{2}$$

where *r_i_*(*t*) is the particle displacement at time *t*; *r_i_*(0) is the initial particle displacement; *N* is the number of particles.

**Figure 11:**
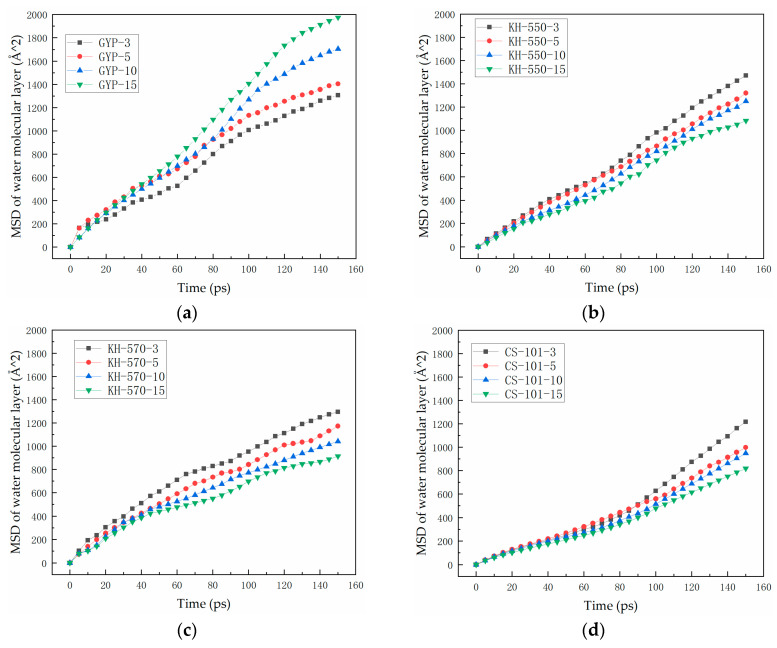
MSD of different types of PAM: (**a**) MSD of PAM with unmodified GYP; (**b**) MSD of PAM with KH-550; (**c**) MSD of PAM with KK-570; (**d**) MSD of PAM with CS-101.

The diffusion extent of the water molecule layer was assessed using qualitative and quantitative methods. The results of the analysis of all control groups were summarised based on Section S3 in the Supporting Information and presented in Figure 11. The nomenclature conventions for the model control groups were derived from Figure 10.

As can be seen from Figure 11, the degree of diffusion of the water molecule layer in the phosphogypsum asphalt mastic–water–mineral aggregate system increases with the increase of phosphogypsum dosing when no coupling agent is added, indicating that the degree of thermal movement of water molecules is increased due to phosphogypsum dosing, which in turn suggests that phosphogypsum dosing can result in the asphalt–aggregate adhesion being affected negatively, further corroborating the fact that unmodified phosphogypsum filler can cause an increase in the water sensitivity of asphalt. Meanwhile, by comparing Figure 11b–d, it is evident that the incorporation of three types of coupling agents leads to a decrease in the diffusion degree of water molecule layer with an increase in the coupling agent-modified phosphogypsum filler. The hydroxylated sulfate ions became more polar while the molecular mass of the phosphogypsum filler increased, resulting in a significant increase in the degree of agglomeration of the high-molecular-weight components within the asphalt’s molecular structure. This, in turn, boosted the adsorption of water molecules and mineral aggregates by the asphalt mastic layer.

**Figure 12:**
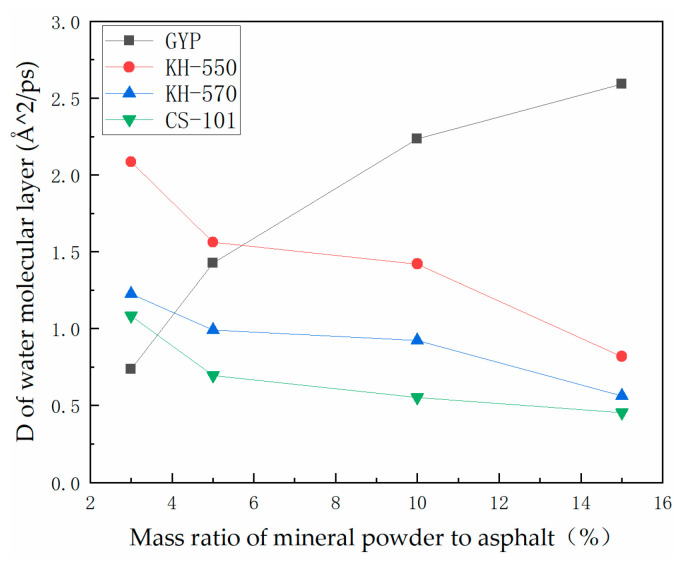
Diffusion coefficient of different types of PAM.

Upon previous analysis, it is evident that there exists a distinct difference in the effect of the three coupling agents on the water stability of asphalt–aggregate. In the case of KH-550, there seems to be no correlation between the MSD trend and MRPA when the latter is at low levels. However, once the MRPA of KH-550 increases to 10–15% and the simulation time goes beyond 120 ps, the trend of an augmented degree of water molecular layer diffusion gradually decelerates. There is no significant difference in the water stability modification effect of phosphogypsum asphalt mortar at various dosages for KH-570. However, the effect is superior to that of KH-550 at equivalent dosages. When MRPA reaches 15%, its trend is similar to that of KH-550-15 after exceeding 120 ps of simulation time. For KH-570, the water stability modification effect of phosphogypsum–asphalt mortar under different dosages is not significant, but it is better than that of KH-550 under the corresponding dosage. As the dosage of CS-101-modified phosphogypsum increases, the MSD of water molecules in the layer exhibits a consistent trend with simulation time, with the displacement trend becoming increasingly uniform. This results in greater consistency and uniformity of water molecule displacement within the layer. When the CS-101 dosage reaches 15%, the water molecules’ mean square displacement in the entire simulation time appears to reduce slightly. This could be attributed to the larger relative molecular mass of CS-101, which has a significantly longer molecular chain compared to KH-550 and KH-570. Moreover, its reaction products with phosphogypsum minerals are anticipated to develop spatial entanglement or group clustering with the asphalt’s molecular structure. Additionally, to quantitatively compare and analyse the impact of various coupling agent-modified phosphogypsum on the water sensitivity of asphalt mastic–aggregate, the diffusion coefficients generated from the MSD results are consolidated and graphed in Figure 12. Using Equation (4), the particle diffusion coefficients are computed.

(4) $$D=\frac{1}{6}\underset{t\to \infty }{\lim}\frac{d}{dt}\sum_{i=1}^{N}{\left[r_{i}\left(t\right)-r_{i}\left(0\right)\right]}^{2}$$

where *D* is the diffusion coefficient of the particle.

Figure 12 illustrates that when no coupling agent is used in phosphogypsum asphalt mastic, the diffusion coefficient of water molecules in the laminar system comprising asphalt, mastic, water, and aggregate increases with the increase in the mass ratio of mineral powder to the asphalt. The findings suggest that the addition of phosphogypsum significantly improves the diffusion degree of the water molecule layer when the coupling agent is not doped. Furthermore, the phosphogypsum increases the water sensitivity of asphalt. Consequently, the adhesion of asphalt to the aggregate becomes more susceptible to the intervention of water molecules on the asphalt–aggregate interface. For the modified phosphogypsum asphalt mastic with KH-550, there is a noticeable decline in the water molecule layer’s diffusion coefficient with an increase in the mass ratio of modified mineral powder to asphalt. The results indicate that the addition of KH-550 coupling agent to the modified phosphogypsum decreases the water sensitivity of the asphalt with increasing dosage. However, the water sensitivity of the asphalt decreases when the dosage of the modified phosphogypsum is increased, compared to the dosage without the coupling agent. When the coupling agent is introduced, the diffusion coefficient of the water molecule layer in the KH-550-modified model is greater than that in the model lacking the coupling agent at an MRPA of 3–5% to the asphalt. It is preliminarily deducible that the combination of KH-550 and phosphogypsum does not significantly restrict the diffusion of water molecules in lower content. The agglomeration of components with larger molecular weights in asphalt will only be significantly restrained when the content is increased. For KH-570, the diffusion coefficient of the water molecule layer decreases at a slower rate with the increase of the mass ratio of modified mineral powder to asphalt. This means that the diffusion coefficient of the water molecule layer is relatively low when the mass ratio of modified mineral powder to asphalt is low. However, it is greater than the control group of the asphalt mastic model with unmodified phosphogypsum at 3%. This suggests that the modification mechanism of water sensitivity in phosphogypsum is alike for KH-570 and KH-550, as well as the modification mechanism of water sensitivity in asphalt mastic being similar for KH-570 and KH-550. The modification mechanisms of KH-570 and KH-550 in phosphogypsum on the water sensitivity of asphalt mastic exhibit similarities; however, KH-570 yields a superior overall modification effect than KH-550. A comparison of their structural characteristics reveals that increasing the length of a carbon chain in a coupling agent can effectively enhance the modification of its water stability.

## 4. Discussion

Based on the analysis above, results from Figure 11 showed that the reaction between the coupling agent and calcium sulfate hemihydrate in phosphogypsum alters the molecular structure of the pristine calcium sulfate hemihydrate. The organic long carbon chains grafted on the hydroxylated sulfate ions exhibited unique structural features that reduced the sensitivity of the pristine hydroxylated sulfate ions to water.

Figure 12 shows that there is a significant decrease in the diffusion coefficient of the water molecule layer in the asphalt mortar–water–aggregate system of CS-101 PAM when the mass ratio of mineral powder to asphalt increases. Furthermore, as the dosage grows from 3% to 15%, this decreasing trend remains, with its reduction rate being gradually diminished. At an MRPA of 3%, the constraints on the diffusion of water molecules are lower than those in the unmodified PAM. The PAM molecule system usually contains only about two CS-101-modified phosphogypsum molecules due to the large relative molecular mass of CS-101 and the limitation of the model calculation volume. The positioning of molecules within the asphalt system may impact their ability to modify water sensitivity. As the quantity of modified CS-101 molecules escalates, their distribution in the asphalt cell model becomes more uniform. This consequentially results in a greater effect on the overall polarity of the PAM molecular model.

Based on molecular dynamics simulation, a molecular model of coupling agent-modified phosphogypsum asphalt mortar–water–mineral aggregate was established, and the process of interfacial adhesion between asphalt and mineral aggregate was investigated by simulating different water content conditions, and the diffusion behaviour of water at the adhesion interface was analysed, which clarifies the mechanism of the coupling agent-modified phosphogypsum on the optimization of interfacial adhesion enhancement. The main discussions are as follows:

- The hydroxylated sulfate ions in phosphogypsum show significant hydrophilicity, and the diffusion rate of its small molecules in asphalt is faster, which leads to the existence of a water film and thus reduces the adhesion between asphalt and aggregate. After coupling agent modification, the hydrophilic group of sulfates is transformed into the long carbon chain of the coupling agent, which leads to the enhancement of the molecular polarity characteristics. This process may produce the phenomenon of the asphaltene molecules entangled in the aggregation, thus promoting the adsorption of asphalt and aggregate mineral components.
- In the absence of a coupling agent, the addition of phosphogypsum causes diffusion of the water molecule layer in the asphalt mortar model. Because of phosphogypsum, the water sensitivity of asphalt increases, resulting in greater water molecule intervention on the asphalt–aggregate interface. Increasing the length of the carbon chain of the coupling agent can indirectly enhance the modification effect of the coupling agent on water stability. The limiting effect of KH-570 on interfacial water is stronger than that of KH-550.
- The diffusion coefficient of the water molecular layer of asphalt mastic modified with CS-101 coupling agent in phosphogypsum shows a significant decrease with the increase of CS-101-modified phosphogypsum (more than 5% mass ratio to asphalt). Compared with the KH coupling agent, the inhibition effect of CS-101 coupling agent on water diffusion is more significant. However, when these coupling agents are adopted in phosphogypsum-modified asphalt (with lower mass ratio to asphalt), it is preferable to use a KH type coupling agent when considering water stability.
- From the results of this study, it can be concluded that when the coupling agent-modified phosphogypsum is used as mineral powder filler (with higher mass ratio to asphalt), the coupling agent modification can significantly restrict the diffusion of water molecules and improve the adhesion performance of PAM, in which CS-101 coupling agent has the best effect, followed by KH-570 and KH-550.
- The modelling and findings in this paper provide a basis for further investigation of other properties of PAM such as mechanical properties, rheological properties, low temperature properties and self-healing properties. In addition, the research in this paper is mainly based on molecular simulation techniques, and experimental studies for the adhesion of PAM and aggregates and other influencing factors of adhesion are also not fully covered in the existing studies. Further studies will focus on the above-mentioned ideas.

## Figures and Tables

**Table 1:** Composition of AAA-1 molecular asphalt model.

Component			Numbers	Mass Ratio
Types	Name	Formula		
Asphaltenes	Asphaltene-phenol	C_42_H_54_O	3	5.3
	Asphaltene-pyrrole	C_66_H_81_N	2	5.5
	Asphaltene-thiophene	C_51_H_62_S	3	6.6
Resins	Quinolinohopane	C_40_H_59_N	4	5.8
	Thioisorenieratane	C_40_H_60_S	4	7.1
	Benzobisbenzothiophene	C_18_H_10_S_2_	15	13.5
	Pyridinohopane	C_36_H_57_N	4	6.2
	Trimethybenzeneoxane	C_29_H_50_O	5	6.4
Saturates	Squalane	C_30_H_62_	4	5.2
	Hopane	C_35_H_62_	4	6.0
Polycyclic aromatics	PHPN	C_35_H_44_	11	15.8
	DOCHN	C_30_H_46_	13	16.3

## Data Availability

Data are contained within the article and Supplementary Materials.

## Supplementary Materials

The following supporting information can be downloaded at: https://www.mdpi.com/article/10.3390/polym15224412/s1, Section S1: Model Construction Procedures; Section S2: Model Calculation Operation; Section S3: MSD Result Calculation.
