# Supplementary material for: Interfacial Water Stability between Modified Phosphogypsum Asphalt Mortar and Aggregate Based on Molecular Dynamics
Source: Polymers (Basel). 2023 Nov 15;15(22):4412. doi: 10.3390/polym15224412 (PMC10675047; doi:10.3390/polym15224412)
Supplement: Supplementary file 1 [file polymers-15-04412-s001.zip › polymers-2666454-supplementary.pdf]

Supplementary Material

S1. Model Construction Procedures

1. Matrix Asphalt Components

The preliminary model construction is carried out with a target density of 0.75g/cm3, and then "Quench" calculation is carried out under the Constant temperature, constant pressure (NPT) system. In the Quench trajectory file, the research object for subsequent optimization calculation is the single frame model with the closest density to that of the laboratory matrix asphalt [18-20]. Then, 5000 iterations of geometric optimization were carried out, utilizing the optimized asphalt model as the asphalt molecular model in the interface MD model.

2. Phosphogypsum molecular model:

The main steps taken were:

Select the "Build" toolbar label in MS software, further select "Nanocluster" in Nanostructure, select the type as "Sphere", set the radius to 5 Ångström (Å,1 Å=10-10m), and set other options to default values.

Click "Build" to generate an initial calcium sulfate hemihydrate nanoclusters. Before the modification of hemihydrate calcium sulphate by silane coupling agent, the usual process is to use sodium sulphate and sodium hydroxide solution mixed into hemihydrate calcium sulphate to achieve its hydroxylation[22]. So, hydrogenation treatment is applied for the initial calcium sulfate hemihydrate model.

3. Phosphogypsum mortar molecular model

Based on the three modified product molecular models displayed in Figure 6, the construction of an adjusted phosphogypsum mortar molecular model primarily comprises the subsequent stages:

Following the ratio of the number of molecules listed in Table 1 and utilizing the Condensed-phase Optimized Molecular Potentials for Atomistic Simulation Studies version III (COMPASS III) force field, the molecular models for each component were entered as component models of amorphous crystal cells. Furthermore, the molecular models of the three coupling agents modified products were added proportionally to achieve the target density of 0.8g/cm^3.

The Quench and geometric optimization on the original asphalt model were performed. The NPT ensemble, a temperature of 298.15K, a simulation interval of 1fs, and a length of 100ps were selected for the Quench. Besides, the output model frequency was one model every 2000 steps, and all other simulation parameters were set to default values. According to the statistical table generated by the data calculations, the density outcomes of the model utilising the Quench calculation table were examined, and the asphalt model with a conclusive density above 1.02 ± 0.005g/cm^3 was elected.

4. Interface Model between PAM Mortar and Aggregate Mineral Model

Based on the construction foundation of various molecular models mentioned above, via "Build layer" function of MS, aggregate mineral molecular models, water molecular models, and phosphogypsum filler asphalt mortar models modified with different coupling agents were respectively added to the layered structure, and then an asphalt mortar water aggregate interface model considering different coupling agent modified phosphogypsum fillers and filler content is established, which is constructed as a crystal model.

After preliminary construction, each model undergoes 5000 iterations of geometric optimization, and the molecules situated below 10 Å on the surface of the quartz aggregate model are set as constraint states via the "Constraints" function. This approach aims to accurately reach the relaxed surface of the mineral aggregates and the stable internal crystal structure.

S2. Model Calculation Operation

After carefully assessing the model's scale and parameter settings from relevant research, we used the "Forcite" module to conduct dynamic calculations by applying the NVT ensemble at 298.15K while implementing a time step of 1fs and simulation time of 150ps. The simulation used default values for all parameters, with the exception of "Fine" calculation accuracy. The results of dynamic simulation for all control group models were obtained. After completion of the layered interface model calculation, the aggregate, water film and asphalt mortar components in the cell model system should be independently removed for each model. It is necessary to calculate the energy of each individual component of the asphalt and mineral system using the same parameter settings. Each control group of models, comprising dynamic equilibrium calculations of individual water molecular layers, must be processed similarly to obtain changes in energy before and after constructing the layered model. Additionally, the energy variation pattern at the interface should be analysed.

S3. MSD result calculation

Further analyses were conducted to compare and evaluate the impact of phosphogypsum on the water sensitivity of the asphalt-aggregate interface in the absence and presence of various coupling agents. Trajectory files from kinetic calculations were used for these calculations. The initial step involved the selection of the water molecule layer and the calculation of its corresponding centre of mass using the “Centroid” function in the toolbar. Subsequently, the movement of the water molecule layer in several control groups was further assessed using the MSD function in the “Analysis” of “Forcite” module. Finally, the overall degree of diffusion of the water molecule layer was evaluated through a combination of qualitative and quantitative techniques.
